# Dynamics of Individual Red Blood Cells Under Shear Flow: A Way to Discriminate Deformability Alterations

**DOI:** 10.3389/fphys.2021.775584

**Published:** 2022-01-05

**Authors:** Scott Atwell, Catherine Badens, Anne Charrier, Emmanuèle Helfer, Annie Viallat

**Affiliations:** ^1^Aix Marseille Univ, CNRS, CINAM, Marseille, France; ^2^Aix Marseille Univ, INSERM, MMG, Marseille, France; ^3^APHM Service de Génétique Médicale, Centre de référence pour la Drépanocytose, les Thalassémies et les maladies constitutives du Globule Rouge et de l'Erythropoïèse, Hôpital de la Timone, Marseille, France

**Keywords:** RBC under shear flow, RBC deformability, RBC density, sickle cell disease, flip-flopping/tank-treading transition

## Abstract

In this work, we compared the dynamics of motion in a linear shear flow of individual red blood cells (RBCs) from healthy and pathological donors (Sickle Cell Disease (SCD) or Sickle Cell-β-thalassemia) and of low and high densities, in a suspending medium of higher viscosity. In these conditions, at lower shear rates, biconcave discocyte-shaped RBCs present an unsteady flip-flopping motion, where the cell axis of symmetry rotates in the shear plane, rocking to and fro between an orbital angle ±ϕ observed when the cell is on its edge. We show that the evolution of ϕ depends solely on RBC density for healthy RBCs, with denser RBCs displaying lower ϕ values than the lighter ones. Typically, at a shear stress of 0.08 Pa, ϕ has values of 82 and 72° for RBCs with average densities of 1.097 and 1.115, respectively. Surprisingly, we show that SCD RBCs display the same ϕ-evolution as healthy RBCs of same density, showing that the flip-flopping behavior is unaffected by the SCD pathology. When the shear stress is increased further (above 0.1 Pa), healthy RBCs start going through a transition to a fluid-like motion, called tank-treading, where the RBC has a quasi-constant orientation relatively to the flow and the membrane rotates around the center of mass of the cell. This transition occurs at higher shear stresses (above 0.2 Pa) for denser cells. This shift toward higher stresses is even more remarkable in the case of SCD RBCs, showing that the transition to the tank-treading regime is highly dependent on the SCD pathology. Indeed, at a shear stress of 0.2 Pa, for RBCs with a density of 1.097, 100% of healthy RBCs have transited to the tank-treading regime vs. less than 50% SCD RBCs. We correlate the observed differences in dynamics to the alterations of RBC mechanical properties with regard to density and SCD pathology reported in the literature. Our results suggest that it might be possible to develop simple non-invasive assays for diagnosis purpose based on the RBC motion in shear flow and relying on this millifluidic approach.

## Introduction

The high deformability of red blood cells (RBCs) is an essential element of blood fluidity. It plays a role in the coupling between RBCs and flow and, therefore, in their individual dynamics, such as shape and orientation in the flow. Cellular deformability also modulates RBC interactions with blood vessel walls and blood cells, contributing to the margination phenomenon of rigid leukocytes and platelets in large vessels, or acting on cell clustering in thin capillaries. A decrease in RBC deformability is observed in a number of diseases, such as diabetes mellitus, sickle cell disease, or malaria, and is postulated as a major determinant of impaired blood microcirculation (Yedgar et al., 2002; Baskurt and Meiselman, 2003). Indeed, reduced cell deformability alters the ability of RBCs to change their overall shape to pass through the smallest capillaries of the microcirculation and the inter-endothelial slits in the spleen (Salehyar and Zhu, 2016; Gambhire et al., 2017; Safeukui et al., 2018). Also, in sickle cell disease where RBC deformability decreases and their stickiness increases, the combined alterations potentially lead to thrombus formation and vaso-occlusive events in capillaries. It is therefore important to be able to detect alterations of RBC deformability in health and disease.

The deformability of RBCs is controlled by their cellular mechanical properties. The first mechanical parameter is the shear elasticity of the cell membrane, which controls the local membrane deformations at constant surface area. The higher the shear elasticity the more difficult it is to deform the membrane. The two other parameters are the membrane and the cytoplasm viscosities, which are responsible for the dynamics of deformation and the associated energy dissipation in the membrane and the cytoplasm, respectively. The higher the viscosity, the more energy is dissipated when a flow is created in the membrane and in the cytoplasm. These three mechanical properties drive the cell motion under shear flow. Indeed, RBCs exhibit several types of motion, from tumbling like a coin, rolling like a wheel, and tank-treading like a droplet, depending on the values of their mechanical properties, the applied shear flow, and the viscosity of the suspending fluid.

**Figure 1:**
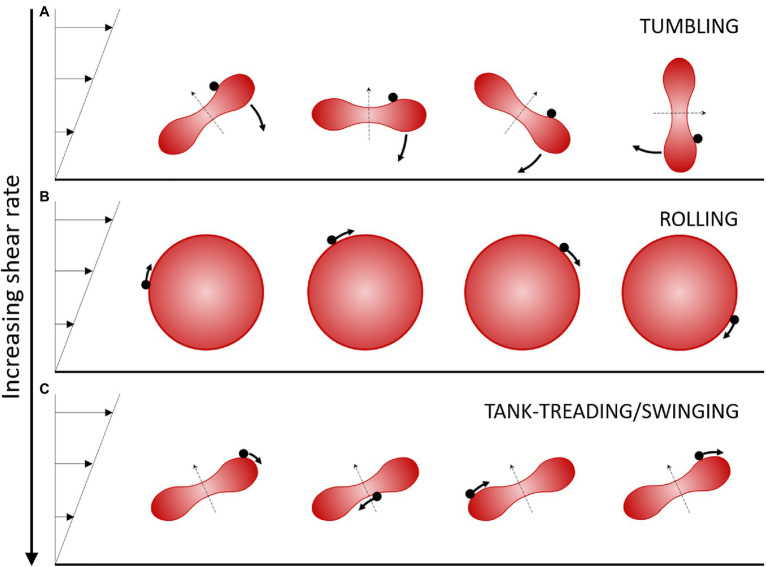
Schematic view of the regimes of motion of a discocyte-shaped RBC in moderate shear flow. Flow is from left to right. The shear stress is increased from **(A)** to **(C)**. The RBC is illustrated over time perpendicularly to the shear plane. The black dot on the RBC displays a membrane element in motion. The thin dashed arrow indicates the axis of revolution of the RBC. **(A)** The tumbling/flip-flopping motion is observed at very low shear stress (typically <0.05–0.1 Pa for healthy RBCs). **(B)** The rolling motion is observed at intermediate shear stresses (typically 0.1–0.15 Pa for healthy RBCs). The axis of symmetry is perpendicular to the shear plane. **(C)** The tank-treading/swinging motion is observed at higher shear stress (typically >0.15 Pa for healthy RBCs). The axis of symmetry remains in the shear plane and the body of the RBC oscillates slightly around a fixed tilted position as the membrane rotates around it.

A flow condition for which several different RBC motions are successively observed upon increasing the shear rate over a small range (1 to 10 s^−1^) is when RBCs are suspended in a medium of “high” viscosity, typically 20 to 40 times the water viscosity. In these ranges of shear rate and external medium viscosity, the shear stress exerted on the RBCs remains small (less than 0.4 Pa) so that they do not undergo strong deformations and keep their global physiological biconcave disk shape. However, the local shear strains of the cell membrane and the viscous dissipation in the cytoplasm are very sensitive to the applied external stress, which leads to different types of cell motion when the shear rate is increased at a given viscosity (Dupire et al., 2012). More precisely, for an external fluid of viscosity 20–40 times the water viscosity, at very low shear rate, healthy RBCs tumble in the flow like a rigid body, and their axis of symmetry lies in the shear plane (Figure 1A). When the shear rate increases, RBCs progressively change their orientation to display a complex flip-flopping motion with a precession motion of their axis of symmetry. This motion is also characteristic of a rigid-like motion but the axis of symmetry of the cell does not lie in the shear plane. Upon further increase of the shear rate, RBCs reach a stable regime of rolling, also a rigid-like motion, in which the cell spins in the shear plane and rolls on its edge like a wheel (Figure 1B). In this motion, the axis of symmetry of the RBC is perpendicular to the shear plane. Finally, when the shear rate is increased above a critical value which depends on the viscosity ratio between the RBC viscosity and that of the external fluid (typically >0.15 Pa for an external viscosity 40 times that of water), RBCs present a droplet-like behavior. The membrane tank-treads, that is, it rotates around the center of mass of the cells, and the RBC orientation oscillates (swings) around a mean value (Fischer et al., 1978; Abkarian et al., 2007; Figure 1C). The axis of symmetry of the cell lies in the shear plane.

The intimate relationship between the values of the three mechanical parameters and the observed types of motion at a given shear stress have generated numerous experimental (Schmid-Schönbein and Wells, 1969; Goldsmith and Marlow, 1972; Fischer et al., 1978; Bitbol, 1986; Abkarian et al., 2007; Dupire et al., 2012; Fischer and Korzeniewski, 2015) and numerical studies (Abkarian et al., 2007; Skotheim and Secomb, 2007; Fedosov et al., 2010; Yazdani and Bagchi, 2011; Cordasco et al., 2014; Peng et al., 2014; Sinha and Graham, 2015; Mauer et al., 2018; Mendez and Abkarian, 2018; Mignon and Mendez, 2021). It is today clear that the shear elasticity of the membrane controls the rigid-like motions (tumbling, flip-flopping, and rolling), that is, the precession motion (Abkarian et al., 2007; Dupire et al., 2012). At a given shear stress, the higher the shear modulus, the closer to tumbling the cell motion is. Indeed, RBCs with a stiffened membrane only tumble and do not roll. It is also clear that the transition to tank-treading is a transition from a rigid to a fluid object (Abkarian et al., 2007; Dupire et al., 2012; Mauer et al., 2018). It therefore strongly involves the values of both membrane and cytoplasm viscosities.

The approaches above, whether experimental or numerical, have mainly focused on healthy RBCs. The cell behavior under flow is expected to change under pathological conditions which alter the RBC deformability, such as sickle cell disease (SCD), a prevalent and very handicapping genetic disease. SCD is an inherited blood disorder caused by sickle hemoglobin (HbS), a variant of the hemoglobin (Hb) molecule resulting from a single nucleotide variation in the β-globin gene. Upon deoxygenation, HbS polymerizes and self-assembles into fibers in the cytoplasm, resulting in sickled-shaped cells and causing abnormal blood rheology (Chien et al., 1970). Upon re-oxygenation, most RBCs regain a discocyte shape, the remaining ones keep an irreversible sickle shape (ISCs) and are among the densest RBCs (Noguchi and Schechter, 1981; Noguchi et al., 1983). Whatever their shape and density, RBCs from SCD patients display a cytoplasmic viscosity higher than healthy ones (Byun et al., 2012). Non-ISCs exhibit a shear elasticity in the range of healthy cells both at large and low deformation regimes (Evans et al., 1984; Nash et al., 1984; Byun et al., 2012). However, the denser ISCs display a higher membrane shear modulus (Byun et al., 2012). Table 1 presents a summary of healthy and SCD (non-ISCs) RBC mechanical parameters, measured in whole blood or density-sorted RBCs (Charache et al., 1967; Chien et al., 1970; Linderkamp and Meiselman, 1982; Evans et al., 1984; Nash et al., 1984; Byun et al., 2012). Additionally, the experimental flow studies have been carried out on whole populations of healthy RBCs, without considering intra-population differences. RBCs in the blood are actually of different age, and older RBCs are known to be denser and to display modified mechanical properties. The RBC behavior under shear flow can thus also be affected by density.

**Table 1:** Mechanical properties of healthy and SCD RBCs reported in the literature, measured on either unsorted (whole) or density-sorted (light and dense) RBCs with different techniques (micropipette, membrane fluctuations, and rheometer).

		Healthy		SCD (non-ISCs)	
Whole	μ η_m_ η_cyto_	5–9 μN/m^[1–4]^ 0.7–0.8 μN.s/m^[1,2]^ 5–10 mPa.s^[3,6,7]^		ns^[2]^, ns^[4]^ ns^[2]^, ns^[4]^ +325%^[3]^	**≈** **≈** **+++**
Light (MCHC ≤33 g/dl)	μ η_m_ η_cyto_	ns^[1]^, ns^[4]^ −25%^[1]^ at least −10%^[6,7]^	**≈** ─ ─	ns^[2]^, ns^[3]^, ns^[4]^ ns^[2]^ +220%^[3]^	**≈** **≈** **++**
Dense (MCHC ≥36 g/dl)	μ η_m_ η_cyto_	ns^[1]^, ns^[4]^ +30%^[1]^ at least +50%^[6,7]^	**≈** **+** **+**	ns^[2]^, ns^[3]^, ns^[4]^ +100%^[2]^ +360%^[3]^	**≈** **++** **+++**

*μ*: membrane elastic shear modulus; *η*﻿_m_: membrane viscosity; *η*_cyto_: cytoplasm viscosity. Typical range values are given for unsorted healthy RBCs. The properties measured for sorted healthy and SCD RBCs are compared to those of unsorted healthy RBCs: the relative changes are given for each reference (ns: non-significant), and the global tendencies are indicated as **≈** (sensibly equal), ─ (less) or + (greater). For clarity, the specific references are additionally numbered in the table and listed below: [1] Linderkamp and Meiselman, 1982. [2] Nash et al., 1984. [3] Byun et al., 2012. [4] Evans et al., 1984. [5] Charache et al., 1967. [6] Chien et al., 1970.

In this work, we used a millifluidic chamber to create a linear shear flow at the vicinity of the wall to compare homozygous SCD RBCs, compound heterozygotes for sickle cell and beta-thalassemia RBCs, and healthy RBCs, all sorted by density, in oxygenated condition. By characterizing the cell motion as a function of the shear rate at a given external viscosity, we aimed at understanding which mechanical parameters are altered for populations of RBCs of various densities, and for RBCS from SCD patients. In contrast with previous studies that focused on ISCs, thus only present in SCD samples, we investigated here the behavior of discocyte-shaped RBCs, present in all types of samples. We refined our observations on SCD cells with the density parameter which is being closely related to the morphological characteristics and deformability of SCD cells (Evans et al., 1984). We correlate the observed behaviors with specific alterations of the cellular mechanical parameters and highlight the role of cytoplasmic and membrane viscosities.

## Materials and Methods

### Blood Samples

Blood specimens are a subset of residual samples referred to the Department of genetics (Hôpital de La Timone, Marseille, France) for routine tests (hematological parameters and age of donors are given in Table 2). Blood was obtained from three healthy donors (HbAA), three homozygous subjects with SCD (HbSS), and one subject with Sickle Cell-β-thalassemia (HbβS). HbSS patients were non-transfused and selected preferentially with low fetal Hb (HbF) fraction (7–18%). Blood samples were mixed with EDTA when harvested at hospital and stored at 4°C. Samples were retrieved less than 24 h after harvesting and RBCs were immediately density-sorted (see the corresponding paragraph below) before final resuspension in SAG-mannitol (SAGM, EFS, France) at 50% hematocrit. RBC samples were then stored at 4°C and used within 1 week.

**Table 2:** Age and hematological data of donors.

Patient	Age (years)	% HbS	% HbF	% HbA0	Hct (L/L)	MCV (μm^3^)	MCH (pg/cell)	MCHC (g/dL)
Range for healthy RBCs	N/A	N/A	N/A	N/A	0.37–0.47	80–98	27–32	30–36.5
HbAA 1	39	0	0.8	85	0.39	88	30	34
HbAA 2	47	0	0.5	86.1	0.42	82.5	28.6	34.6
HbAA 3	35	0	0.5	86	0.42	86	30	34.4
HbSS 1	22	84	7.1	2.1	0.21	81.6	29.8	36.5
HbSS 2	33	72.4	18.1	1.8	0.19	101	38	37.6
HbSS 3	32	71	11.3	10.5	0.18	91.5	32.3	35.3
HbβS	10	72	9.9	7.2	0.24	69.9	23.2	33.2

Patients are named after their genetic mutations: HbAA for homozygous healthy patients without mutations, HbSS for homozygous SCD and Hb*β*S for Sickle Cell-*β*-thalassemia patients. Hematological data were measured at the hospital with an automated hematology analyzer (Sysmex XN-10, Sysmex CorporationTM, Japan). % HbS, % HbF, and % HbA0 are the measured percentages in S-mutated, fetal, and non-mutated hemoglobin, respectively. Hematocrit (Hct), Mean Corpuscular Volume (MCV), Mean Corpuscular Hemoglobin (MCH), and Mean Corpuscular Hemoglobin Concentration (MCHC) are given for each sample.

### Buffers

Dulbecco’s Phosphate Buffered Saline (DPBS 1X, 14190, Gibco) was adjusted to pH 7.4, and to an osmolarity of 295 ± 5 mOsm using an osmometer (Gonotec OSMOMAT 030) by adding glucose. Dextran (from Leuconostoc mesenteroides, ≈2000 kDa, D5376, Sigma-Aldrich) was solubilized at 9% (wt/wt) in PBS by overnight stirring at 50°C. Dextran solution had a viscosity *η_o_* = 39.2 ± 0.7 10^−3^ Pa·s at room temperature, measured with a cone-plane rheometer (MARSIII Cone C35/2°, Thermo Fisher/Haake) and its density matched RBC density, thus preventing cell sedimentation.

### RBC Density Sorting and Estimation of Relative RBC Content in Each Layer

**Figure 2:**
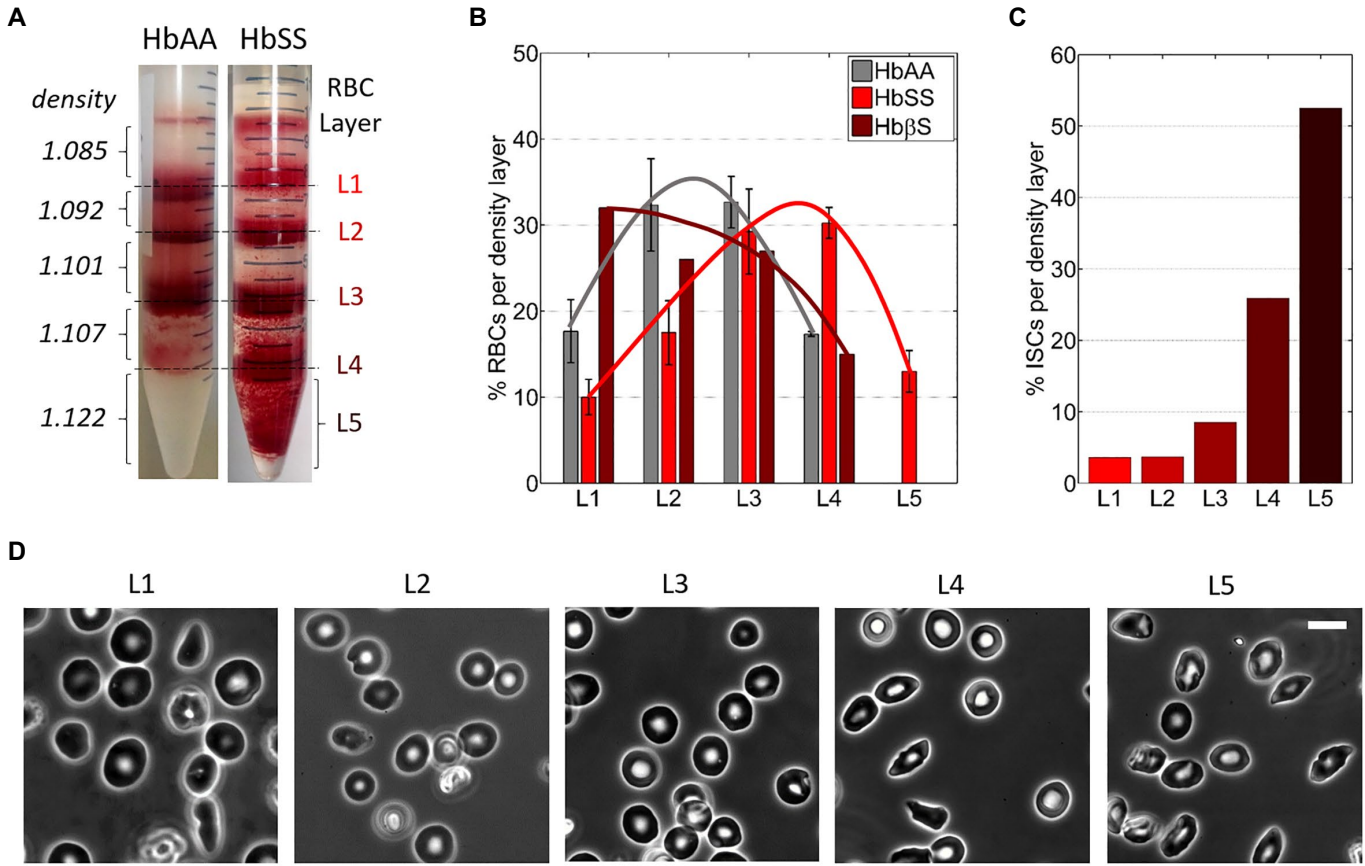
RBCs from Sickle Cell Disease patients are denser. **(A)** Typical images of RBC layers from healthy (HbAA) and SCD (HbSS) blood samples after sorting in discontinuous Percoll gradients of increasing densities *d*. **(B)** Relative content in RBCs for the five density layers in various blood samples: HbAA (averaged over three samples); HbSS (averaged over three samples); HbβS (one sample). The lines are guides to the eyes. **(C)** Percentage of irreversible sickle RBCs (ISCs) per density layer for an HbSS patient (calculated over ≈300 RBCs per density layer). **(D)** Typical 100× phase-contrast images of RBCs in each of the five layers in the same HbSS patient. Scale bar: 10 μm.

Percoll density gradient was used to separate blood samples right after collection into RBC density fractions. Percoll (P1644, Sigma-Aldrich) was diluted in water adjusted to 300 mOsm and pH 7.4 to densities of 1.085, 1.092, 1.101, 1.107, and 1.122 g/ml as in Corbett et al. (1995). The blood sample from hospital was diluted in PBS and centrifuged at 500 *g* for 10 min at 4°C; then, the RBC pellet was resuspended in PBS at 50% hematocrit. The Percoll solutions (2 ml of each) were layered on top of each other in a 15-ml centrifuge tube, from the denser to the lighter one. A 2-ml volume of the RBC suspension was layered on top of the Percoll gradient and centrifuged at 4000 *g* for 1 h at 4°C. RBCs were redistributed as a function of their respective density into four fractions at the interfaces between the Percoll layers (with average densities of 1.0885, 1.0965, 1.1040, and 1.1145 g/ml, respectively) and a denser fraction in or below the densest Percoll layer (with a density ≥ 1.122 g/ml; Figure 2A). The five fractions were harvested from the gradient using a Pasteur pipette, from top to bottom, and then processed for storage: three consecutive washing steps (two in PBS and a third one in SAGM) followed by final resuspension in SAGM. The RBC density fractions were stored as described above.

The relative content in RBCs for each density layer was estimated from pictures of the centrifugation tubes after sorting (Figure 2A). The images were acquired with a 1 Mpixels digital color camera and processed using Matlab. Only the red channel of the RGB images was kept and converted to grayscale; then, the layers were delimitated manually, based on intensity. The signal intensity was then integrated on the height of each layer, following the assumption that RBC density is linearly correlated to the signal intensity. The minimum signal was baselined using the top portion of the gradient devoid of RBCs. The percentage of each layer was then calculated as the ratio of the integration result for the layer divided by the sum of results for all layers. This evaluation, while not fully quantitative, gives a rough estimate of the density distribution of RBCs in blood samples (Figure 2B).

The percentage of irreversible sickle cells (ISCs) in each density layer was calculated from phase-contrast microscopy images of RBCs from each layer (≈300 cells/layer): ISCs were identified *via* their elongated non-discocytic shape.

### Flow Experiments and Microscopy

**Figure 3:**
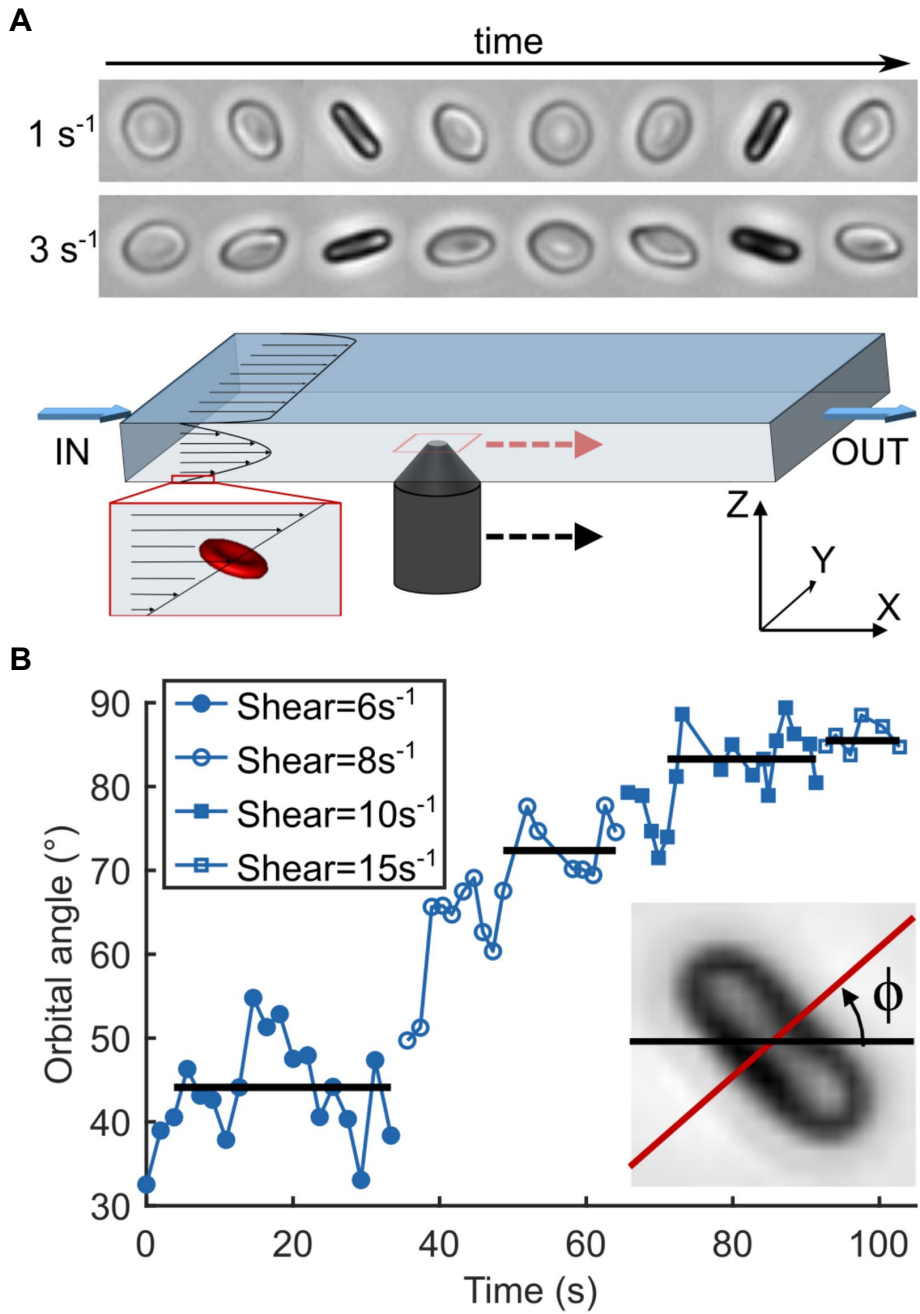
Observation of RBC motion as a function of increasing shear rate. **(A)** Schematics of the experimental device. A viscous dextran solution containing RBCs is pushed through a parallelepiped chamber (L × W × H: 50 × 10 × 1 mm^3^). Individual cells of discocyte shape are chosen near the bottom wall (≤50 μm) and away from the sides where vertical shear is linear and the horizontal shear is null. They are observed along the flow gradient (*Z*-axis) and followed manually while flowing through the chamber by moving the objective. Timelapses on the top show the motion cycles of the same healthy RBC undergoing tumbling at shear rates of 1 and 3 s^−1^ with orbital angles of 35 and 75°, respectively. **(B)** Temporal evolution of the orbital angle ϕ of an SCD RBC as a function of a step-by-step increasing shear rate. ϕ is the angle between the orthogonal projection of the cell axis of revolution along the flow gradient (*Z*-axis) and the flow direction (*X*-axis). Horizontal lines correspond to stabilized ϕ values for each shear rate. Inset: Measurement of ϕ on an RBC image. It is measured each time the cell is on the edge, that is, when the cell axis of symmetry is perpendicular to the direction of observation.

Flow experiments were performed as described previously (Dupire et al., 2012; Figure 3A). Briefly, stored RBCs were diluted (≈500×) in dextran solution and injected in a parallelepiped quartz flow chamber (50 × 10 × 1 mm^3^) mounted on an inverted microscope (DMIRB, Leica) equipped with a 20× objective and a Photonfocus camera. The fluid was driven by a syringe pump (11 Plus, Harvard Apparatus) at wall shear rates $\dot{\gamma }$ ranging from 1 to 20 s^−1^ (i.e., shear stresses $\eta_{o}\dot{\gamma }$ ranging from 0.04 to 0.8 Pa). RBCs were observed within 50 μm from the bottom wall (zone of constant shear rate) in brightfield microscopy along the direction of the flow gradient (Z-axis in Figure 3A). Discocyte-shaped RBCs were selected and tracked individually along the flow direction by manually moving the stage to keep the RBC in the center of the field of view. During video acquisition, the shear rate was increased step by step when the flip-flopping RBC displayed a stabilized orbital angle at a given shear rate. Images were recorded at 25 fps and then processed semi-automatically using Matlab routines to measure the orbital angle of the individual RBCs.

### Data Analysis

For each sample (L2 and L4 density layers from blood samples) RBCs were tracked at typically 3 to 5 shear rate values between 1 and 20 s^−1^. In healthy and SCD blood samples, at least 14 RBCs were tracked in each layer while only 12 RBCs were tracked in L2/L4 layers of the Sickle Cell-β-thalassemia blood sample. For a given shear rate, the median value within a sample was calculated from orbital angles measured for all tracked RBCs that were still in the flip-flopping regime. As the number of RBCs still in the flip-flopping regime gradually decreased when the shear rate increased, we arbitrarily defined a minimum threshold of 4 RBCs per shear rate per sample to calculate the ϕ median value. The ϕ-standard deviation per sample was also calculated at the shear rate of 0.04 Pa (between 4 and 18 flip-flopping RBCs per sample).

## Results

### RBCs Densities and Morphologies Are Consistent With the Literature

RBC fractions of increasing density sorted at the Percoll interfaces were referred to as layer 1 to layer 4 (L1 to L4), respectively (Figure 2A). RBCs harvested in or below the densest Percoll layer (1.122 g/ml) were referred to as layer 5 (L5; Figure 2A, right). The top layer above the 1.085 g/ml Percoll layer was discarded as it mainly contained RBC fragments.

Figure 2B shows the estimated average layer partition of the RBC content for HbAA, HbSS, and HbβS samples (non-averaged data in Supplementary Figure 1). The RBC distribution in HbSS samples was shifted toward denser fractions as compared to HbAA samples and the densest L5 layer (d_RBC_ ≥ 1.122) was present only in HbSS samples, as classically reported in the literature (Kaul et al., 1983; Embury et al., 1984; Fabry et al., 1984; Mohandas et al., 1989; Kumar et al., 2014). The RBC distribution of the HbβS sample displayed no L5 layer and spread toward lighter fractions. Consistently, the MCHC measured on the HbβS sample is the lowest among the seven blood samples (see Table 2), thus disclosing an Hb deficit within individual Sickle Cell-β-thalassemia RBCs. We estimated the mean density of each blood sample by averaging the percentages of RBCs present in each layer weighted by the average density of the layer. This estimated mean density correlated well with the MCHC measured on each blood sample, as displayed in Supplementary Figure 2 together with data collected from the literature, thus validating our approach to estimate the RBC amount in the density layers.

The percentage of irreversible sickle cells (ISCs) per layer, distinguishable *via* their elongated shape, is shown in Figure 2C for one HbSS sample. Typical associated cell morphologies from the five layers are illustrated in Figure 2D. In agreement with previous studies (Kaul et al., 1983), the percentage of ISCs in a given layer increased with the layer density, up to more than 50% in L5.

### The Flip-Flopping Behavior at Low Shear Stress Is Similar for SCD and Healthy RBCs

We performed flow experiments as previously described (Dupire et al., 2012) to compare the behavior of healthy and SCD individual RBCs suspended in a medium of viscosity *η_o_* = 39 Pa·s under increasing shear flow (Figure 3). Only discocyte-shaped cells were selected in order to test whether SCD RBCs of normal appearance displayed altered dynamics. In our previous study on healthy RBCs (Dupire et al., 2012), the cells were unsorted and, though behaving qualitatively similarly, data were scattered between individual cells, probably due to random density. As such dispersion could hinder the detection of altered behavior we sorted the RBCs in density and compared the motion of cells from healthy and SCD populations of same density. We selected the layers L2 and L4, as both are present in the two types of blood samples and almost reversed in RBC proportion (32.3 ± 3.7% for L2 and 17.3 ± 0.3% for L4 for HbAA samples vs. 17.5 ± 3.8% for L2 and 30.3 ± 1.8% for L4 for HbSS samples). Additionally, the two layers are also present in the Sickle Cell-β-thalassemia sample.

**Figure 4:**
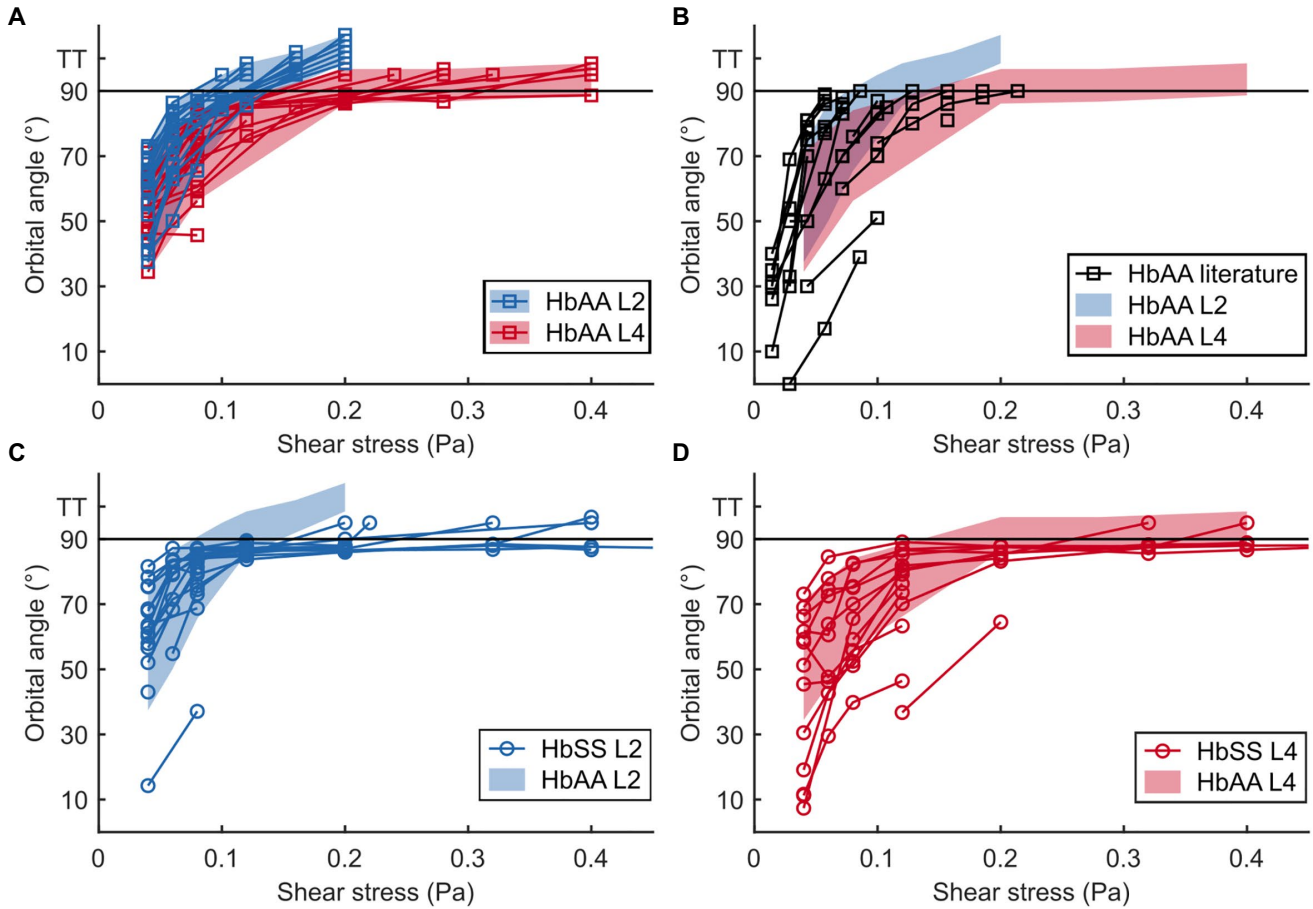
The flip-flopping regime is similar in healthy and SCD samples but the transition to tank-treading regime is delayed for SCD RBCs. **(A,B)** Evolution of the orbital angle ϕ vs. shear stress $\eta_{0}\dot{\gamma }$ for L2 and L4 layers of a healthy sample (HbAA; **A**) and comparison with data found in literature **(B)**. L2 and L4 data in **(A)** are highlighted with color-shaded regions (blue and red, respectively) to facilitate comparison with other data sets. **(C,D**) Orbital angles for L2 **(C)** and L4 **(D)** layers of an SCD sample (HbSS) compared to those of the HbAA shown in **(A)**. At least 14 RBCs were tracked in each sample. For all samples, tank-treading RBCs (TT, no ϕ value) are artificially scattered above the line drawn at 90° for clarity. The tank-treading regime was not characterized in the literature data.

At low shear rate $\dot{\gamma }$ , both healthy and SCD RBCs presented an unsteady periodic flip-flopping motion, close to the tumbling motion, in which they rock to and fro between an angle ±ϕ, called the orbital angle, which depends on the $\dot{\gamma }$ value (see timelapses in Figure 3A). At very low $\dot{\gamma }$ , the orbit of RBCs oriented with ϕ < 30° was usually found to be unstable over the observation time (≈50–100 s; Supplementary Figure 3). Therefore, we increased the shear rate until the chosen cells displayed a stabilized initial ϕ-value between 30 and 40°. For most RBCs, ϕ then progressively increased with the shear rate, eventually reaching almost 90°, that is, the rolling regime where the RBCs lay in the shear plane (Figure 3B). Upon further $\dot{\gamma }$ increase, most RBCs switched to tank-treading motion (displayed as points scattered above the 90° ϕ-value in Figure 4) at a critical cell-dependent $\dot{\gamma }$ value: they oriented their symmetry axis in the shear plane and moved along the flow with a fixed orientation. All these observations are in agreement with those reported by Dupire et al. (2012) and with computational results (Cordasco and Bagchi, 2013; Sinha and Graham, 2015).

**Figure 5:**
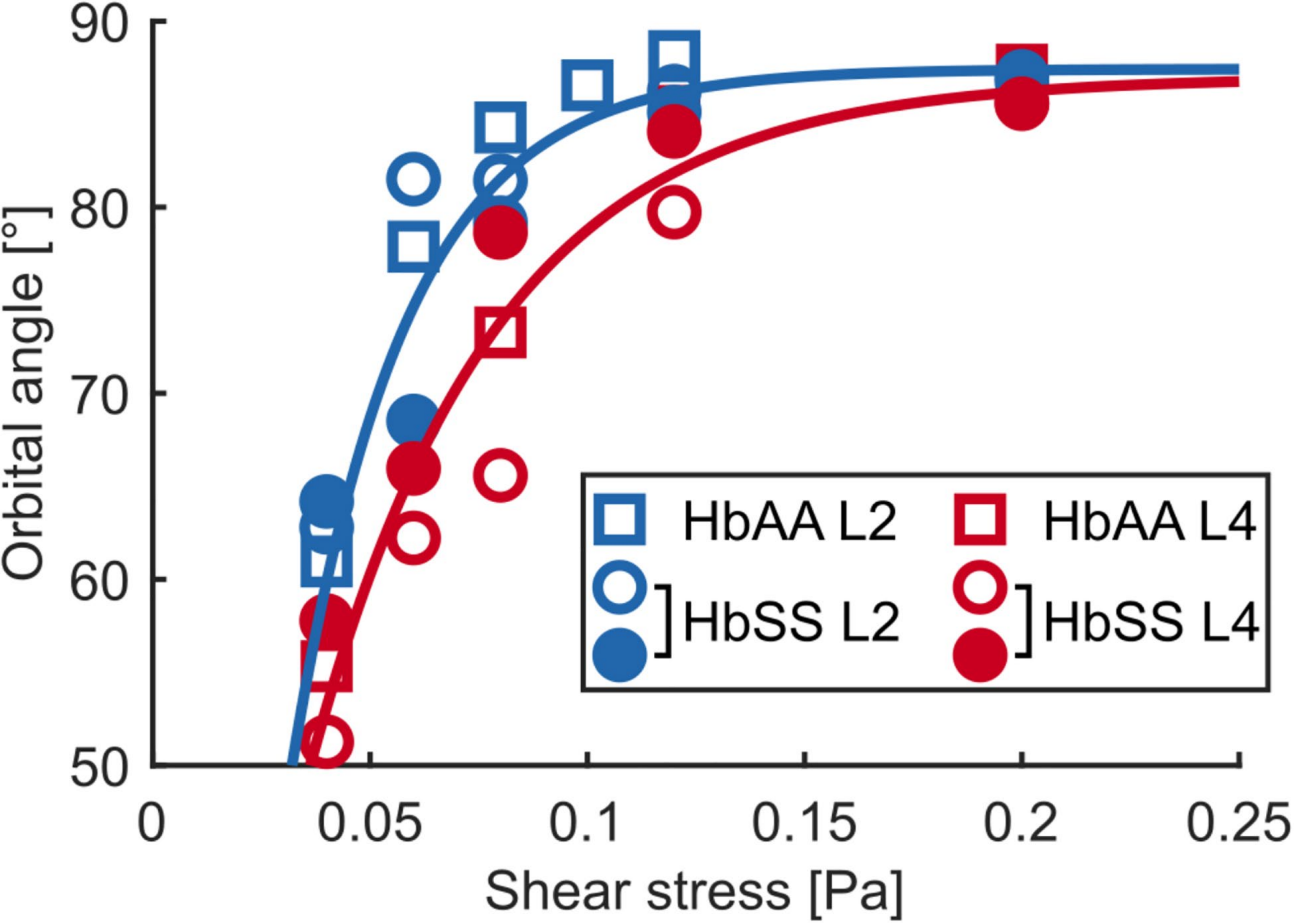
The flip-flopping motion depends on density. Median orbital angle ϕ as a function of shear stress $\eta_{o}\dot{\gamma }$ for L2 and L4 layers of an HbAA sample and of two HbSS samples. The blue and red lines are visual guides for the behaviors of L2 (blue symbols) and L4 (red symbols) RBCs, respectively, regardless of healthy or SCD type.

At low shear stresses (typically $\eta_{o}\dot{\gamma }$ < 0.1–0.15 Pa), for which most RBCs are in the flip-flopping regime, a striking difference was clearly detected between RBCs from L2 and L4 layers. Angles ϕ of lighter RBCs (L2) were observed to increase faster than those of denser RBCs (L4) upon increasing shear stress, regardless of whether they were of healthy or SCD origin (Figures 4A,C,D). Remarkably, mixing the ϕ( $\eta_{o}\dot{\gamma }$ ) data (orbital angle as a function of shear stress) from the L2 and L4 healthy RBCs showed a good overlap with the unsorted data from Dupire et al. (2012; Figure 4B), confirming their dispersion was most likely due to random density variations. Flip-flopping SCD RBCs compared quite well with healthy ones, layer by layer as shown in Figures 4C,D. The layer-to-layer difference is illustrated in Figure 5, where the variations of the median value of ϕ, for one HbAA sample and two HbSS samples, are plotted as a function of the shear stress $\eta_{o}\dot{\gamma }$ for L2 and L4 layers. It clearly shows that healthy and SCD RBCs in the same density range behave closely. SCD RBCs are slightly more disperse though, as confirmed by comparing the standard deviations of the orbital angle at 0.04 Pa shear stress, where all RBCs are in the flip-flopping regime: the standard deviation of both L2 and L4 healthy RBCs was around 10° while it spanned between 15 and 25° for SCD RBCs. Altogether, these observations show that the flip-flopping motion of discocyte-shaped RBCs is sensitive to density rather than to SCD-induced alteration.

Additionally, we analyzed the behavior of RBCs from a Sickle Cell-β-thalassemia blood sample, which yielded very different results (Supplementary Figure 4). The ϕ-evolution with regard to $\eta_{o}\dot{\gamma }$ of L2 HbβS RBCs was much slower than that of L2 HbAA or HbSS RBCs and quite disperse. More strikingly, all RBCs tracked in the L4 layer displayed only flip-flopping motion with low orbital angles, closer to 0°, which did not increase with $\eta_{o}\dot{\gamma }$ up to 0.8 Pa. This behavior is similar to that of rigidified RBCs in shear flow (Dupire et al., 2012).

### SCD RBCs Transit to Tank-Treading Regime at Higher Shear Stress Than Healthy RBCs

**Figure 6:**
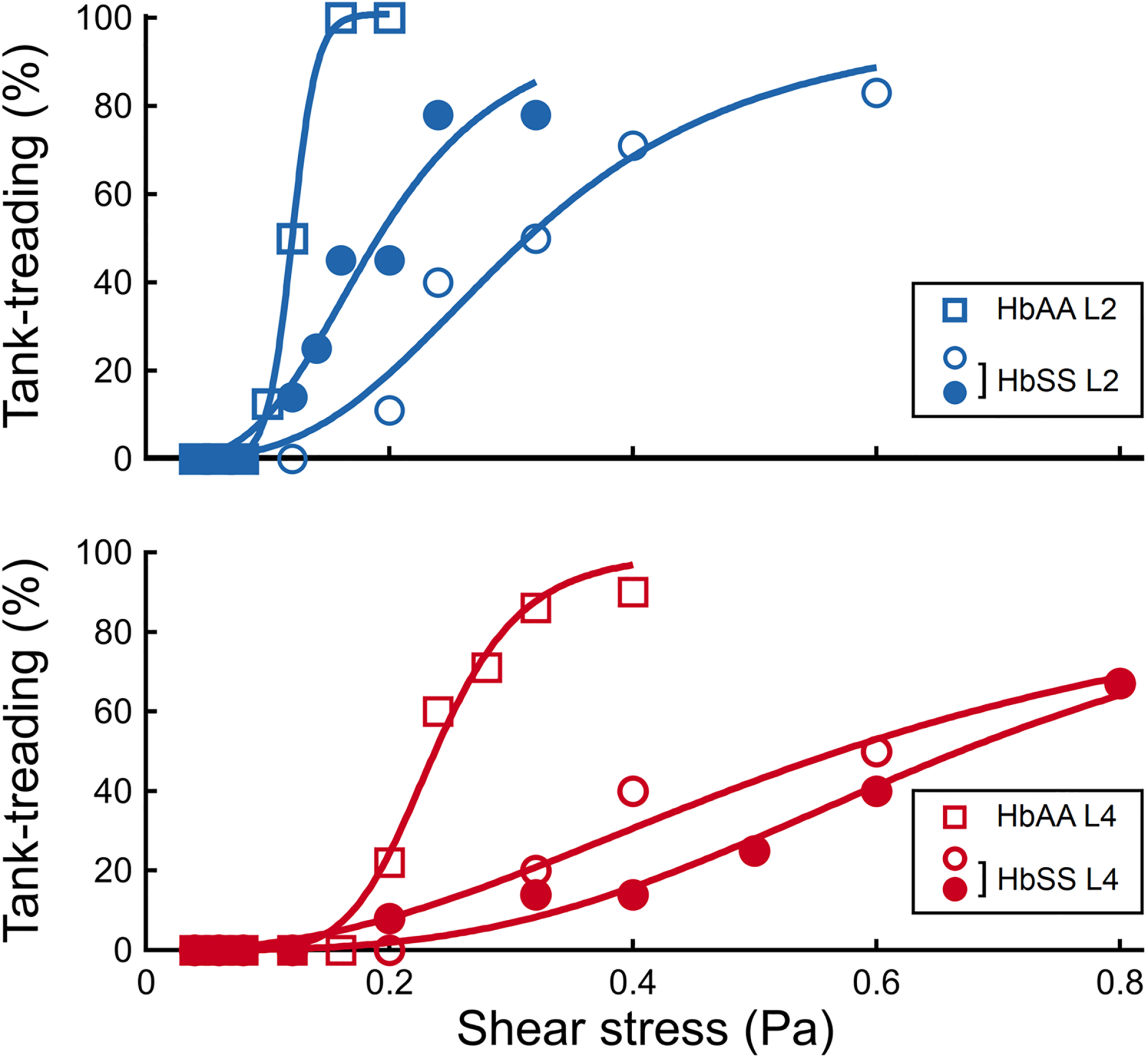
The transition to tank-treading motion is affected by both density and SCD pathology. Percentage of RBCs in tank-treading regime as a function of the shear stress, in the HbAA and HbSS samples, for the L2 and L4 layers, respectively.

It clearly appears from Figure 4A that the transition to tank-treading regime occurred at higher $\eta_{o}\dot{\gamma }$ for healthy RBCs from the L4 layer compared to those from the L2 layer (for clarity, RBCs reaching tank-treading are artificially displayed as having a ϕ-value above 90°). This trend was even more amplified for SCD RBCs, which reached tank-treading at much higher shear stresses than healthy RBCs in each density layer (Figures 4C,D). Accordingly, the percentage of RBCs in tank-treading regime increased slower with shear stress for L4 layers than for L2 ones whether the RBCs are healthy or SCD. Moreover, this percentage remained always lower for SCD RBCs compared to healthy ones of same density (Figure 6). For example, for the L2 layers, at $\eta_{o}\dot{\gamma }$ = 0.2 Pa, all healthy RBCs had reached the tank-treading regime vs. less than 50% of SCD RBCs. This is even more dramatic for the L4 layers: at 0.4 Pa, almost all healthy RBCs were tank-treading vs. less than 40% of SCD RBCs. The delayed transition to tank-treading in SCD RBCs compared to healthy ones is coherent with their slightly lower ϕ $\left(\eta_{o}\dot{\gamma }\right)$ evolution in the flip-flopping regime, which make them reach the transition threshold at higher shear stresses. This delay was even greater for the Sickle Cell-β-thalassemia blood sample, for which only few RBCs from L2 reached tank-treading, at $\eta_{o}\dot{\gamma }$ = 0.6 Pa, and none from L4 (Supplementary Figure 4).

## Discussion

Our study focused on the flow behavior of individual discocyte-shaped RBCs with different deformability. Our flow experiments showed that light and dense RBCs do not behave the same way under the same shear rate and do not transit to tank-treading at the same critical shear rate and that this effect is even higher for SCD RBCs. We seek to explain the observed behaviors with specific changes of the cellular mechanical parameters related to RBC density and SCD nature. Mechanical properties of healthy and SCD RBCs, either unsorted (whole) or density-sorted (light and dense), have been reported in the literature (see Table 1). Note that while these properties were measured mostly on fresh cells, RBC samples were assayed up to 1 week after harvesting in our study. Although this may shift the mechanical parameters, we assume it is similar for all types of RBCs and does not impact our comparative observations. Moreover, our results on healthy RBCs match nicely the data from Dupire et al. who performed the same measurements on fresh blood (Dupire et al., 2012), hence showing no impact of the storage on our observations.

In the non-stationary flip-flopping regime, we showed that the mean RBC orientation in the flow depended on the density of RBCs, regardless of their nature (healthy or SCD). We thus postulate that this observation is solely due to differences in the mechanical properties of light and dense RBCs, and not to SCD pathology. The increase in density of healthy RBCs directly correlates to their aging (Bocci, 1981; Nash and Meiselman, 1981) and was observed to be associated with an increase in both cell cytoplasmic and membrane surface viscosities while the membrane elastic shear modulus remains mostly constant (Table 1). As the shear modulus does not change with density, we naturally assume it would not be the cause of the observed behavior of the orbital angle. The cytoplasmic viscosity increases with density in both healthy and SCD RBCs, with much higher values in SCD RBCs. Since healthy and SCD RBCs behave similarly in the flip-flopping regime, as shown in Figures 4 and 5, we conclude that the cytoplasmic viscosity plays no role in the flip-flopping regime. In fact, the only parameter which changes between the density layers and which is comparable in healthy and SCD RBCs is the membrane viscosity. Thus, we deduce that the behavior differences between RBCs from the L2 and L4 layers are most likely due to variations in their membrane viscosity. The higher dispersion of the $\eta_{o}\dot{\gamma }$ -variations of ϕ for SCD cells may be attributed to the presence of both normal old cells and more rigid pre-ISCs that may already present an increase in their membrane shear elasticity, the latter being undistinguishable in our experiments. To summarize, the difference in the flip-flopping motion between RBCs from L2 and L4 layers is a signature of cell aging, *via* their increase of membrane viscosity only, regardless of the SCD disease.

In contrast, we observed that the $\dot{\gamma }$ -induced transition to tank-treading motion was greatly shifted toward higher values for L4 healthy cells compared to L2 healthy cells. Such results indicate a relation between this transition and density, with the transition happening at higher shear rates for denser RBCs. This relation held for SCD RBCs, but the transition was greatly shifted compared to healthy RBCs, indicating a strong relation between the transition to tank-treading and the nature of the RBC. As above, we now consider which mechanical properties change with density and even more with the SCD nature of the RBCs. The only parameter which varies both with density and between healthy and SCD RBCs is the cytoplasmic viscosity (Table 1). Thus, the higher critical shear rate ${\dot{\gamma }}_{c}$ for which tank-treading transition occurs is attributed to the increase in cytoplasmic viscosity. This is consistent with the role of the cytoplasmic viscosity in RBC transiting from a rigid-like object (in flip-flopping motion) to a fluid droplet (in tank-treading motion) upon increase of the shear flow. The external hydrodynamic flow must overcome the viscous dissipation within the cell, related to cytoplasmic viscosity, to trigger integral fluidization of the RBC and internal circulation of the cytoplasm. Therefore, the tank-treading transition shifted to higher ${\dot{\gamma }}_{c}$ correlates with a higher cytoplasmic viscosity induced by SCD.

The behavior of Sickle Cell-β-thalassemia RBCs was very different from those of healthy and SCD cells, as they do not reach tank-treading. We attribute this to significant cell stiffening as reported in the literature. Indeed, β-thalassemic RBCs were shown to display an increase in both membrane viscosity (Athanassiou et al., 1994) and shear modulus (Athanasiou et al., 1991; De Luca et al., 2008). Membrane shear elasticity is also expected to affect the $\eta_{o}\dot{\gamma }$ -variations of ϕ (Dupire et al., 2012), and computations by Abkarian et al. showed that the critical shear rate ${\dot{\gamma }}_{c}$ is expected to linearly increase with the shear modulus (Abkarian et al., 2007). Consistently, Sickle Cell-β-thalassemia RBCs from the L2 layer hardly reach the transition threshold. Even more, for the L4 layer, the changes due to the disease (increased shear modulus and membrane viscosity) and to the higher density (increased membrane viscosity) result in an almost total cell stiffening, with very low ϕ-values close to those observed upon artificial rigidification with glutaraldehyde (Dupire et al., 2012).

To conclude, the individual behavior of RBCs under shear flow at moderate shear rates is highly informative. The shear stress-related variations of RBC flip-flopping motion allow discriminating between low- and high-density cells that are usually thought as being young and old cells. The critical shear rate at which the transition to tank-treading occurs is sensitive to the cytoplasmic viscosity, thus enabling to distinguish SCD RBC populations from healthy ones. Importantly, we are able to discriminate SCD RBCs in their non-ISC shape, a population similar in shape to healthy RBCs, usually not considered in SCD samples though it is the most abundant. Our results suggest that it might be possible to develop simple non-invasive assays based on the motion of RBCs in shear flow for diagnosis purpose. Such assays, relying on the flip-flopping to tank-treading transition, could be used to discriminate populations of SCD vs. healthy RBCs.

## Data Availability Statement

The original contributions presented in the study are included in the article/**Supplementary Material**; further inquiries can be directed to the corresponding author.

## Ethics Statement

Ethical review and approval was not required for the study on human participants in accordance with the local legislation and institutional requirements. Written informed consent from the participants’ legal guardian/next of kin was not required to participate in this study in accordance with the national legislation and the institutional requirements.

## Author Contributions

CB and AV designed the study. CB provided materials. SA performed the acquisition. SA, AC and EH performed the analysis. SA, EH, and AV drafted the manuscript. All authors contributed to the article and approved the submitted version.

## Funding

This work has been carried out thanks to the support of the A*MIDEX project (n° ANR-11-IDEX-0001-02) funded by the Investissements d’Avenir French Government program, managed by the French National Research Agency (ANR).

## Conflict of Interest

The authors declare that the research was conducted in the absence of any commercial or financial relationships that could be construed as a potential conflict of interest.

## Publisher’s Note

All claims expressed in this article are solely those of the authors and do not necessarily represent those of their affiliated organizations, or those of the publisher, the editors and the reviewers. Any product that may be evaluated in this article, or claim that may be made by its manufacturer, is not guaranteed or endorsed by the publisher.

## Supplementary Material

The Supplementary Material for this article can be found online at: https://www.frontiersin.org/articles/10.3389/fphys.2021. 775584/full#supplementary-material
