# Supplementary material for: Dynamics of Individual Red Blood Cells Under Shear Flow: A Way to Discriminate Deformability Alterations
Source: Front Physiol. 2022 Jan 5;12:775584. doi: 10.3389/fphys.2021.775584 (PMC8767062; doi:10.3389/fphys.2021.775584)
Supplement: Supplementary file 1 [file Data_Sheet_1.pdf]

SUPPLEMENTARY MATERIAL

Dynamics of individual red blood cells under shear flow: a way to discriminate deformability alterations

Scott Atwell, Catherine Badens, Anne Charrier, Emmanuèle Helfer*, and Annie Viallat

* Correspondence:
Emmanuèle Helfer, emmanuelle.helfer@univ-amu.fr

Supplementary Figures

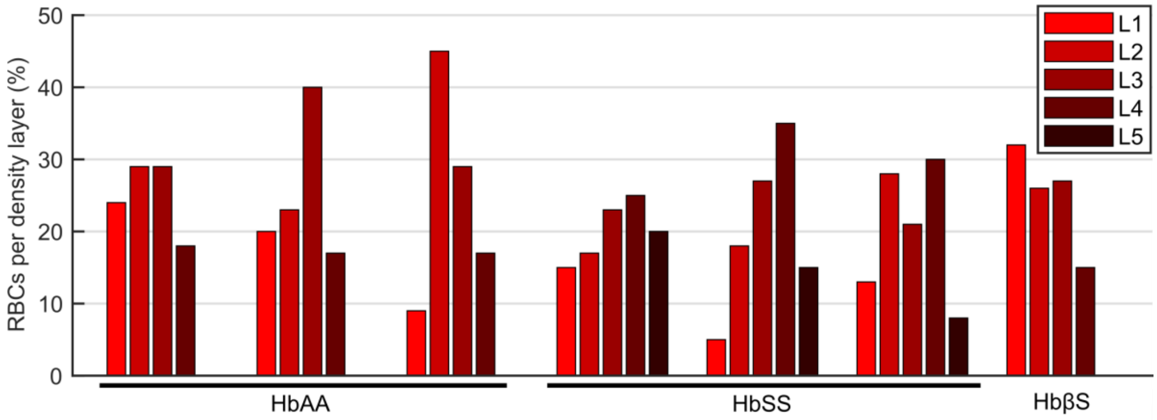

Supplementary Figure 1. RBC layer content from the seven blood samples: healthy (HbAA), SCD (HbSS), and Sickle Cell-β-thalassemia (HbβS) (non-averaged data from Figure 1).

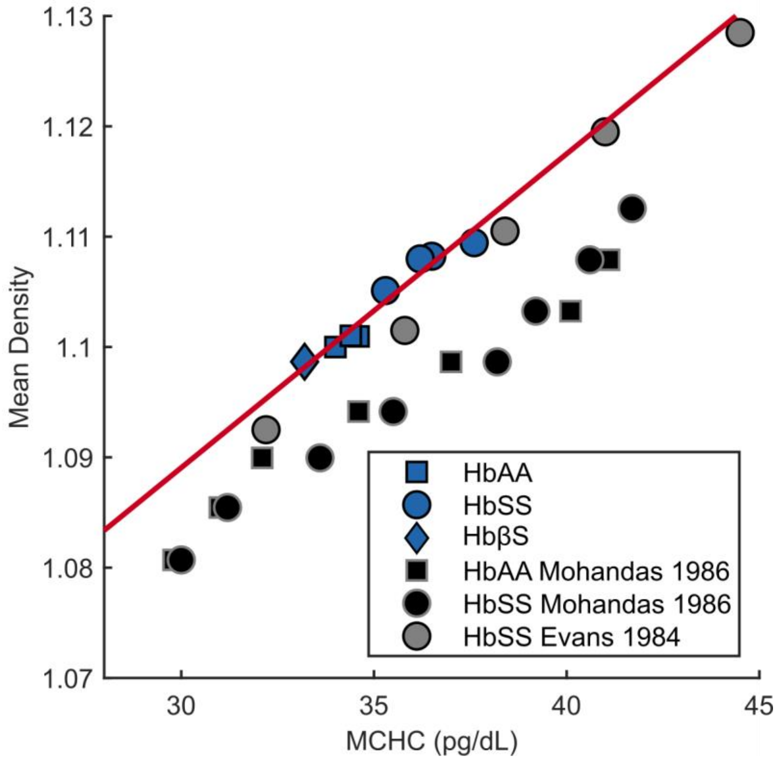

Supplementary Figure 2. Blood sample mean density as a function of mean corpuscular haemoglobin volume (MCHC). Blue symbols: mean density estimated from the RBC distributions among the density layers in Fig. S1. Black and grey symbols: data from the literature (Mohandas et al., Blood (1986); Evans et al. Blood (1984)).

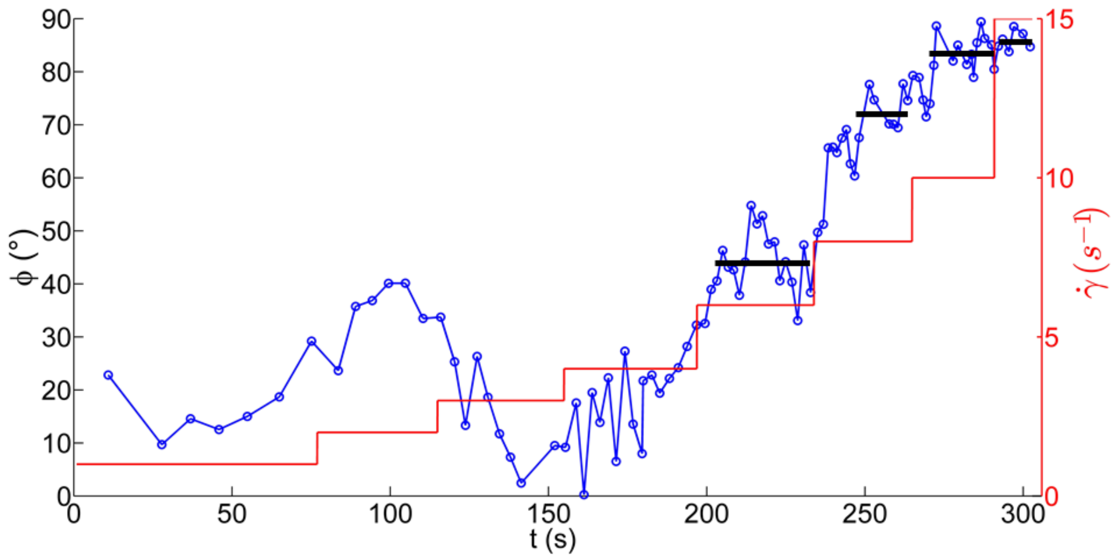

Supplementary Figure 3. Typical behaviour of the orbital angle φ as function of the shear rate $\dot{\gamma}$. At low $\dot{\gamma}$, below 6 s^{-1} here, φ is usually unstable. The black lines indicate stabilized φ.

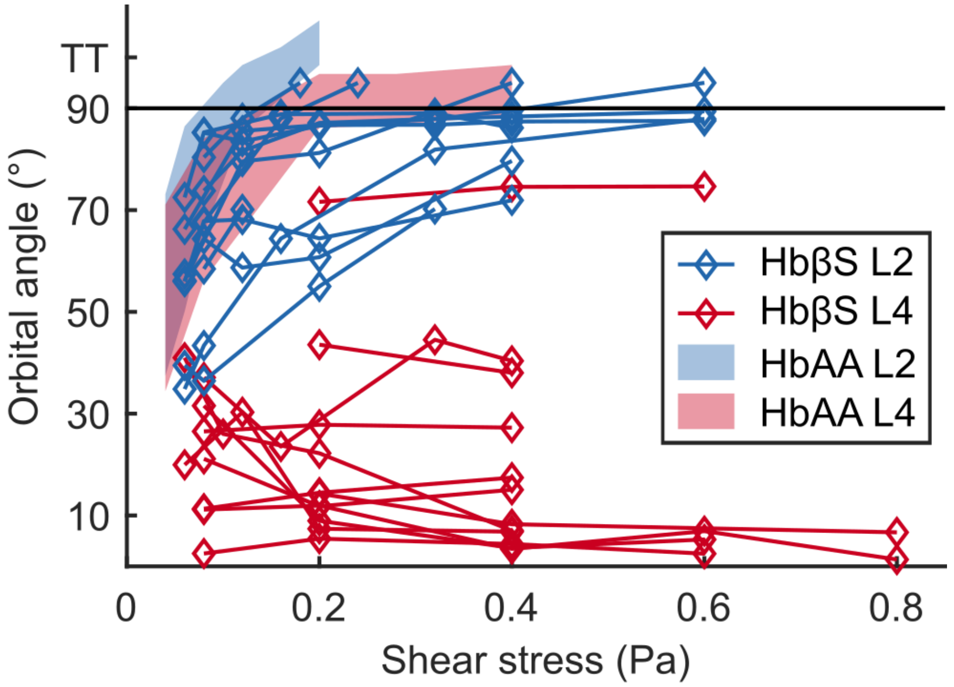

Supplementary Figure 4. The Sickle Cell-β-thalassemia sample behaves differently from healthy and SCD samples. Evolution of the orbital angle ϕ versus shear stress $\eta_o \dot{\gamma}$ for L2 and L4 layers of the Sickle Cell- β-thalassemia sample (HbβS) (12 RBCs per density sample), compared to HbAA data from Fig. 4A (color-shaded regions).
